# A Nanohelicoidal Nematic Liquid Crystal Formed by a Non‐Linear Duplexed Hexamer

**DOI:** 10.1002/anie.201802881

**Published:** 2018-05-08

**Authors:** Richard J. Mandle, John W. Goodby

**Affiliations:** ^1^ Department of Chemistry University of York York YO10 5DD UK

**Keywords:** liquid crystals, modulated nematics, nematic phase, oligomers

## Abstract

The twist‐bend modulated nematic liquid‐crystal phase exhibits formation of a nanometre‐scale helical pitch in a fluid and spontaneous breaking of mirror symmetry, leading to a quasi‐fluid state composed of chiral domains despite being composed of achiral materials. This phase was only observed for materials with two or more mesogenic units, the manner of attachment between which is always linear. Non‐linear oligomers with a H‐shaped hexamesogen are now found to exhibit both nematic and twist‐bend modulated nematic phases. This shatters the assumption that a linear sequence of mesogenic units is a prerequisite for this phase, and points to this state of matter being exhibited by a wider range of self‐assembling structures than was previously envisaged. These results support the double helix model of the TB phase as opposed to the simple heliconical model. This new class of materials could act as low‐molecular‐weight surrogates for cross‐linked liquid‐crystalline elastomers.

The phenomenon of spontaneous breaking of mirror symmetry manifests in a wide range of scientific disciplines and ongoing problems, from subatomic physics to autocatalysis to biological homochirality.^1^ The twist‐bend modulated nematic phase (TB), predicted by Dozov,^2^ possesses a helical structure with a pitch length of approximately 10 nm;^3^ this phase is chiral despite being typically formed by achiral molecules and is the first example of spontaneous symmetry breaking in a liquid system without accompanying positional order. The TB phase was first identified in liquid‐crystalline dimers,^4^ and the relationship between molecular structure and the incidence of this phase has been an active area of research, with the majority of explored structural variations presented in Figure 1 a.^5^ Apart from being found in low‐molecular‐weight dimers, the generation of the TB phase has been demonstrated in oligomers^6^ and polymers.^7^ The TB phase exhibits a fast (microsecond) electrooptic response^8^ and also provides a simple route to materials with defined nanostructures via in situ photopolymerisation,^9^ which may find use in photonics.^10^

**Figure 1:**
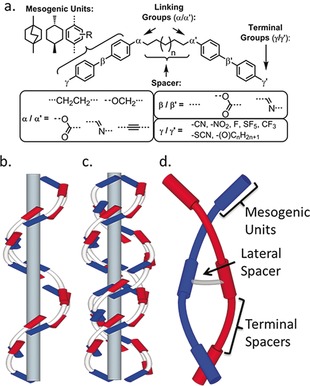
a) General overview of the molecular structures explored within the context of the TB phase. b) Heliconical model proposed by Dozov; c) double twist model suggested by Goodby et al.^11^ and developed by Tuchband et al.;^12^ d) depiction of a duplexed hexamer formed from two laterally appended trimers, with key subdivisions indicated.

Without exception, all examples of the TB phase are found in materials in which the rigid mesogenic units are mutually attached in a linear manner akin to a main‐chain polymer, thereby inducing dimers to behave like polymers. We had suggested that the TB phase may have a double helix structure,^11^ and this idea has been revisited in some detail by others recently.^12^ Such a phase structure, if it exists, should be stabilised by duplexed oligomers in which two (or more) linear oligomers are laterally linked (shown in Figure 1 d), whereas a single helix would be destabilised (Figure 1 b). We devised the novel trimer **D11_3_** and related duplexed hexamer **D11_3_(2)** as a test of this idea.

Suzuki cross‐coupling of ***i1*** with 2,5‐dibromophenol afforded the trimer **D11_3_** (Scheme 1).^13^ Dimerisation of the trimer to afford the hexamer **D11_3_(2)** was achieved by heating **D11_3_** with dibromoheptane and caesium carbonate to 200 °C (sand bath, external temperature) in acetone in a sealed tube (Scheme 1). The structure of **D11_3_(2)** was confirmed by NMR spectroscopy (^1^H and ^13^C{^1^H}) and APCI mass spectrometry, with purity of both the trimer and hexamer assayed by reverse phase HPLC. Computational chemistry was performed in Gaussian G16;^14^ conformer libraries were built via the MODREDUNDANT keyword with geometries and energies extracted via Matlab scripts as described.^15^ Selected output files were visualised using Qutemol.^16^ Full synthetic and instrumental details are given in the Supporting Information.

**Scheme 1:**
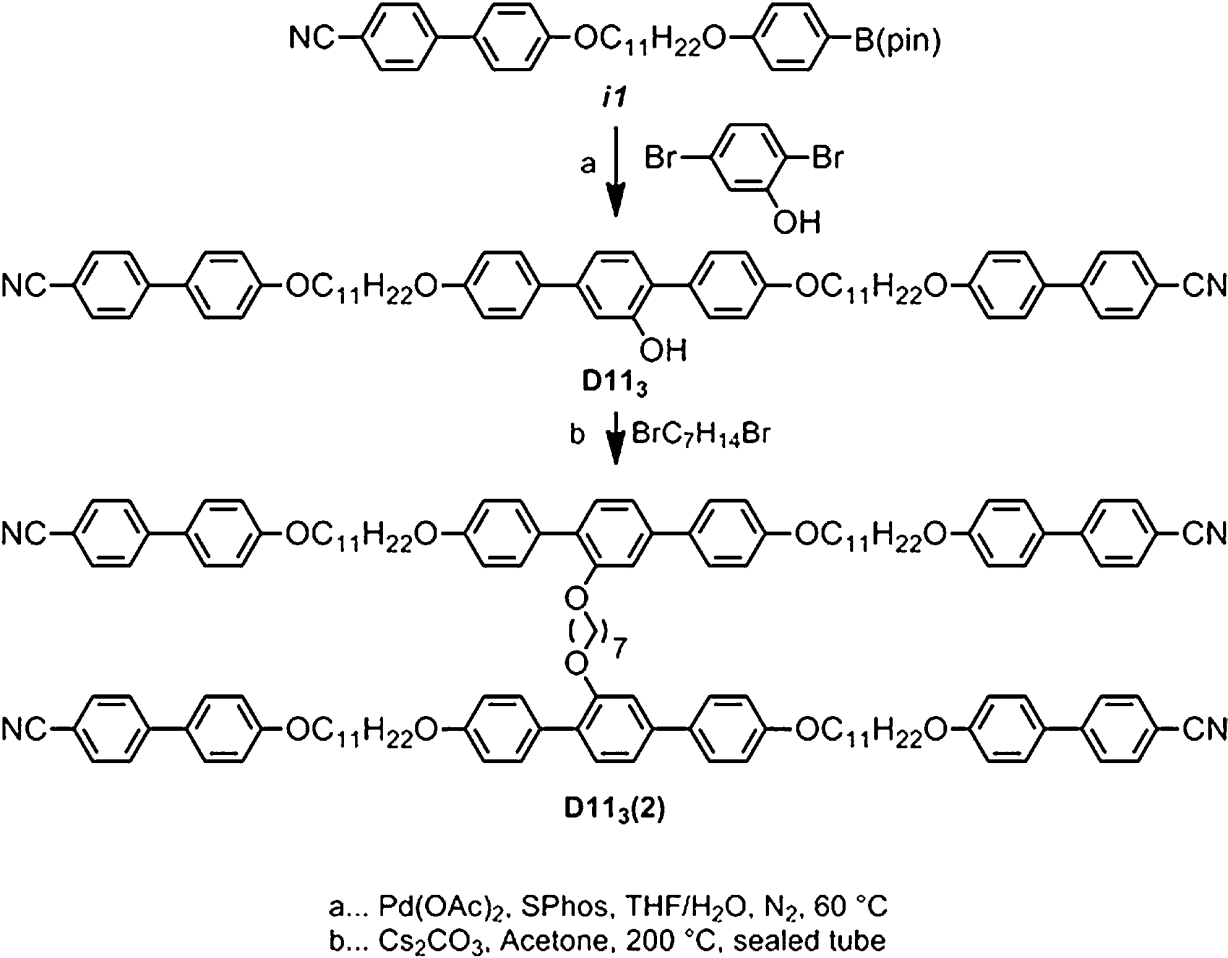
Synthesis of **D11_3_** and **D11_3_(2)** from *i1*.

The trimer **D11_3_** and the hexamer **D11_3_(2)** were studied by polarised optical microscopy (POM) differential scanning calorimetry (DSC) and simultaneous small‐ and wide‐angle X‐ray scattering (SWAXS) to determine transition temperatures and phase types. Tabulated transition temperatures and enthalpies of transition are given in Table 1.

**Table 1:** Transition temperatures *T* [°C] and associated enthalpies of transition Δ*H* [kJ mol^−1^] for the trimer **D11_3_** and derived hexamer **D11_3_(2)**.^[a]^

No.		MP	TB‐N	N‐Iso
**D11_3_**	*T*	125.9	86.3*	172.8
	Δ*H*	32.4	0.6	5.5
**D11_3_(2)**	*T*	123.7	117.2*	172.9
	Δ*H*	93.0	0.5	4.2

[a] Values were obtained by DSC at a heat/cool rate of 10 °C min^−1^ and are the average of 5 cycles. * monotropic phase transition, observed only on cooling below the melting point.

Phase identification was made by a combination of microscopy and SWAXS experiments, whereas transition temperatures and enthalpies were determined from DSC. The nematic phase exhibits a distinctive *schlieren* texture (Figure 2 a), which transforms into the blocky texture immediately following the TB‐N transition (Figure 2 b,c) which evolves into the rope‐like texture with further cooling (Figure 2 d). We confirm these phase assignments for **D11_3_(2)** by way of miscibility with CB9CB,3a a contact preparation shows the two materials are be mutually miscible in both mesophases and therefore our assignment of both phases is correct; photomicrographs are presented in the Supporting Information, Figure S1. Previous results indicate that lateral groups, both polar5b, 6a and non‐polar,^11^ tend to depress the thermal stability of the TB phase; yet dimerisation of the trimer **D11_3_** into the duplex trimer **D11_3_(2)** unexpectedly increases the TB‐N transition temperature by over 30 °C. The enthalpy associated with the TB‐N transition in both materials is comparable to prior (linear) oligomeric examples, and the transition is first‐order.^6^ During SWAXS study no scattering is observed from the TB helix during non‐resonant SWAXS study (Figure 2 f) as has been noted previously,3a although the lack of Bragg scattering supports our assignment as a nematic‐like phase. The intensity of the wide‐angle scattering peak is significantly greater than that at small‐angles, indicating both nematic and TB phases lack significant lamellar fluctuations (cybotaxis). The d‐spacing value of the small angle peak is temperature invariant and has a value of 22.1 Å, this is shorter than the molecular length (see below). The wide‐angle scattering peak has a value of 5 Å, which corresponds to the average lateral molecular separation. We do not observe any further scattering from the sample of **D11_3_(2)** at smaller angles (2*θ*≥0.7°, *q*≥0.5 Å^−1^, *d*≤125 Å); this excludes the possibility of lamellar twist‐bend phases with large layer spacings^17^ and hypothetical splay‐bend modulated nematic phases which should exhibit Bragg scattering at *Q*=$\frac{2\pi }{P{}_{\mathrm{SB}}}$ , where *P*_SB_ is the splay bend modulation period.3a

**Figure 2:**
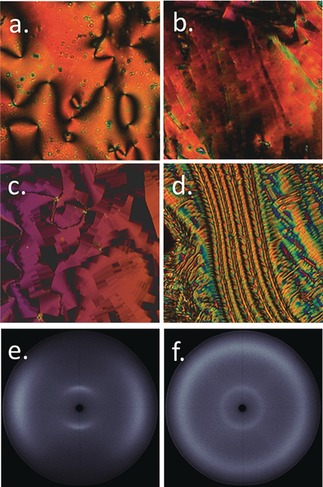
a)–d) Photomicrographs (×100) of: a) the *schlieren* texture of the nematic phase of **D11_3_(2)** at 155 °C, b) the blocky texture of the TB phase of **D11_3_(2)** at 115 °C, c) the blocky texture of the TB phase of **D11_3_** at 82 °C, and d) the rope texture of the TB phase of **D11_3_(2)** at 110 °C; e),f) 2D SWAXS patterns of e) the magnetically aligned nematic phase of **D11_3_(2)** at 160 °C and f) the unaligned TB phase of **D11_3_(2)** at 112 °C. Note that the loss of alignment is spontaneous at the phase transition.

To rationalise SWAXS data we first obtained an optimised, fully extended all‐*trans* structure of **D11_3_(2)_a_** at the B3LYP/6‐31G(d) level of DFT (Figure 3 b). The molecular length of this conformer is 62 Å; taken in conjunction with the d‐spacing of the small angle peak in SWAXS experiments (22.1 Å) this indicates the nematic and TB phases are both extensively intercalated, with no segregation of the different mesogenic units into layers (or pseudo layers) which would lead to Bragg (or quasi Bragg) scattering. The single broad SWAXS peak at small angles is most likely the centre‐to‐centre separation between mesogenic units;^7^ although we do not observe differing scattering peaks for terminally (ca. 24 Å) and laterally (18 Å) appended segments of the molecule. This result is consistent with prior studies on TB forming oligomers, which exhibit small angle scattering at 1/*n* times the molecular length,6b,6c where n is the generation of oligomer (*n*=3, trimer, *n*=4 tetramer, and so on)

**Figure 3:**
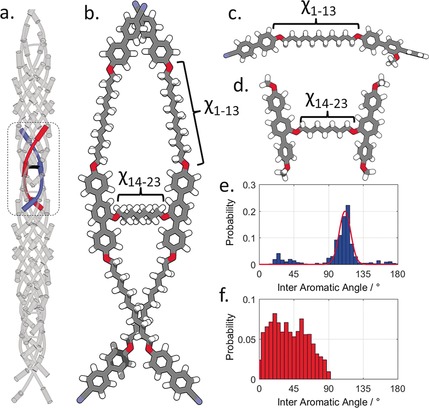
a) Proposed double‐helical structure of the TB phase formed by duplexed hexamers. b) Geometry of **D11_3_(2)** optimised at the B3LYP/6‐31G(d) level of DFT; the dihedrals *χ*_1–13_ and *χ*_14–23_ were used to build conformer libraries of the fragments shown in (c) and (d) at using fully relaxed scans at the B3LYP/6‐31G(d) level of DFT. e),f) Plots of the probability of a given interaromatic bend angle from e) fragment c and f) fragment d. The solid line in (e) is a Gaussian fit to the major peak.

A single geometry neglects the flexibility of this molecule; assuming threefold rotation about each dihedral in the spacer gives an imposing number of conformers (4*(3 13)*3 9), which is too expensive to study computationally. We therefore subdivided **D11_3_(2)** into two fragments, shown in Figure 3 c,d. On each fragment we performed fully relaxed scans at the B3LYP/6‐31G(d) level of DFT, allowing each flexible bond to adopt either *trans* or *gauche* states, giving a library of conformers. Clearly this method ignores intermolecular interactions which could be important in the condensed LC phase, but it provides a useful approximation in this instance. For each conformer we calculate the angle between the two mesogenic units in question and a Boltzmann probability allowing us to present the probability weighted angles given in Figure 3. Within each linear segment the probability of a given bend angle is skewed towards being bent owing to the odd parity of the spacer (Figure 3 e). The major distribution of bend angles is approximately Gaussian, centred at 108° with a FWHM of about 25°. Minor populations of linear (bend >150°) and hairpin (bend <30°) conformers exist.

A broad range of angles are adopted between the two central terphenyl mesogenic units (Figure 3 f); provided these two units are a way off perpendicular (<75°) the formation of a double helical structure is favourable, and we note that there is a decrease in the probability of bend angles over about approximately 60°. If the two central units are (close to) perpendicular then the resulting gross molecular shape would be globular, with the outer nitriles forming the apex of a tetrahedron, rather than a double helix. Presently it is not clear how flexibility (or lack thereof) of this part of the molecule impacts upon TB phase formation.

This conformational study indicates that **D11_3_(2)** is likely to adopt a wide range of conformations, with many of these will being helical or double‐helical structures. If we consider now the parent trimer **D11_3_**, the conformational landscape of this material is effectively defined by the biphenyl–terphenyl bend indicated in Figure 3 d. The formation of a double helix structure by **D11_3_** relies on non‐covalent interactions whereas **D11_3_(2)** forms this structure to covalent bonding of two trimers. The observed enhancement in the thermal stability of the TB phase in **D11_3_(2)** relative to **D11_3_** suggests the double helix structure not only warrants further experimental study, but also suggests that entirely new classes of materials could exhibit this state of matter. We also note the possibility of incorporating stimuli‐ or chemo‐ responsive groups (such as azo,^18^ crown ether) into the lateral spacer to give functional, tuneable, or switchable twist‐bend materials. Just as linear LC dimers and oligomers are considered as good model systems for main‐chain LC polymers,^19^ we consider that the nematic phases of materials such as **D11_3_(2)** could be considered as low‐molecular‐weight surrogates for crosslinked nematic LC elastomers, which have attracted attention as actuators, sensors, and artificial muscle.^20^ Furthermore, the double twisted structure could form a cable‐ or rope‐like arrangement, leading to entirely new forms of matter.

We observe a remarkable stabilisation of the nanohelical TB phase by covalently bonding two trimers together, affording a duplexed hexamer. Previous studies show that lateral substitution at the mesogenic units leads to diminished TB phase stability; however, in the present case the linking of two trimers together actually affords an increase. Rather than forming a single helix the duplexed hexamer is conformationally biased towards double helix formation, and computational studies on the conformational landscape support this idea. We consider that the present results support the double helix model of the TB phase which we had previously proposed,^11^ and has been revisited in more detail by others recently.^12^ There is a need for development of theoretical models of this phase that account for such a helical structure. The observation of the TB phase in a non‐linear oligomer prompts a re‐evaluation of previously held beliefs about the type of molecular structure required to exhibit this nanohelical phase of matter.

## Conflict of interest

The authors declare no conflict of interest.

## Supporting information

As a service to our authors and readers, this journal provides supporting information supplied by the authors. Such materials are peer reviewed and may be re‐organized for online delivery, but are not copy‐edited or typeset. Technical support issues arising from supporting information (other than missing files) should be addressed to the authors.

Supplementary
