# Supplementary material for: A Nanohelicoidal Nematic Liquid Crystal Formed by a Non‐Linear Duplexed Hexamer
Source: Angew Chem Int Ed Engl. 2018 May 8;57(24):7096–100. doi: 10.1002/anie.201802881 (PMC6033141; doi:10.1002/anie.201802881)
Supplement: Supplementary file 1 — Supplementary [file ANIE-57-7096-s001.pdf]

Angewandte Chemie
Eine Zeitschrift der Gesellschaft Deutscher Chemiker

Supporting Information

A Nanohelicoidal Nematic Liquid Crystal Formed by a Non-Linear Duplexed Hexamer

Richard J. Mandle and John W. Goodby*

anie_201802881_sm_miscellaneous_information.pdf

1. Methods

1.1. General Techniques

Reactions were monitored by thin layer chromatography (TLC) using an appropriate solvent system. Silica coated aluminium TLC plates used were purchased from Merck (Kieselgel 60 F-254) and visualised using UV light at wavelengths of both 254 nm and 365 nm. Column chromatography was performed using flash grade silica from Fluorochem (40 - 63μm particle size). Yields refer to chromatographically (HPLC) and spectroscopically (^{1}H NMR and $^{13}C\{^{1}H\}$ NMR) homogenous material.

1.2. Nuclear Magnetic Resonance

NMR spectra were recorded on a JEOL ECS spectrometer operating at 400 MHz (^{1}H), 100.5 MHz ($^{13}C\{^{1}H\}$) as solutions in deuterated chloroform. Spectra were referenced to the residual protic solvent for ^{1}H (7.26 ppm) and $^{13}C\{^{1}H\}$ to the resonance of $CDCl_3$ (77.16 ppm).

1.3. Mass Spectrometry

Mass spectra were recorded on a Bruker compact time of flight mass spectrometer with an APCI source. We extend our gratitude to Mr. Karl Heaton of the University of York for obtaining MS data.

1.4. High Performance Liquid Chromatography

High-performance liquid chromatography was performed on a Shimadzu Prominence modular HPLC system comprising a LC-20A quaternary solvent pump, a DGU-20A$_5$ degasser, a SIL-20A autosampler, a CBM-20A communication bus, a CTO-20A column oven, and a SPO-20A dual wavelength UV-vis detector operating at 220/250 nm. The column used was an Alltech C18 bonded reverse-phase silica column (250 x 4.6 mm) with a 5 μm pore size, an internal diameter of 10 mm and a length of 250 mm and a mobile phase of chloroform/acetonitrile (9:1). Chromatograms with only one peak are quoted at >99.5 % purity.

1.5. Polarised Optical Microscopy

Polarised optical microscopy was performed on a Zeiss Axioskop 40Pol microscope using a Mettler FP82HT hotstage controlled by a Mettler FP90 central processor. Photomicrographs were captured *via* an InfinityX-21 MP digital camera mounted atop the microscope.

1.6. Differential Scanning Calorimetry.

Differential scanning calorimetry was performed on a Mettler DSC822^{e} fitted with an autosampler operating with Mettler Star^{e} software and calibrated before use against an indium standard (onset = 156.55 ± 0.2 °C, ΔH = 28.45 ± 0.40 Jg^{-1}) under an atmosphere of dry nitrogen.

1.7. Computational Chemistry

Quantum chemical calculations were performed using the Gaussian 16 suite of programmes. [1] Output files were visualised in QuteMol. [2] Conformer libraries were constructed using the MODREDUNDANT keyword in Gaussian; a MatLab script was used to read the final geometries and energies of each conformer from the output file and calculate bend-angles and molecular lengths.

2.1. Chemical Characterisation

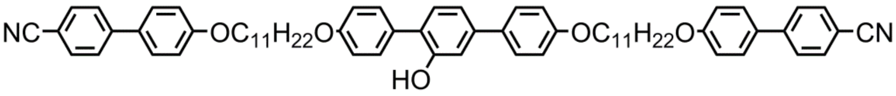

D11$_3$: 4',4'''-((((2'-hydroxy-[1,1':4',1''-terphenyl]-4,4''-diyl)bis(oxy))bis(undecane-11,1-diyl))bis(oxy))bis(([1,1'-biphenyl]-4-carbonitrile))

2,5-Dibromophenol (1 mmol, 252 mg) was added biphasic mixture of THF (10 ml) and saturated aqueous sodium carbonate (4 ml, ~ 2M) which was degassed by sparging with argon in an ultrasonic bath for 10 minutes. Compound ***i1*** (2.1 mmol, 1.2 g) was added in one portion and the degassing continued for 5 minutes. The biphasic mixture was then heated under reflux with vigorous stirring and a nitrogen atmosphere. Seperately, Pd(OAc) (10 mg) and SPHOS (20 mg) were dissolved into deoxygenated THF (2 ml) and vigorously sparged with argon. The Pd-SPHOS solution was added directly to the biphasic reaction mixture which was stirred and heated under reflux for 18 h. The biphasic solution was cooled, the aqueous layer was separated, washed with DCM (3 x 10 ml) and discarded. The combined organic extracts were dried over $MgSO_4$ and concentrated *in vacuo*. The crude material was purified by flash chromatography with DCM as the eluent before recrystalising from sec-butanol/cyclopentylmethylether (40:1) to afford the title compound as a white microcrystalline solid.

Yield: 800 mg (82 %)

Rf (DCM): 0.18 (fluoresces blue when irradiated at 254 & 365 nm)

^{1}H NMR (400 MHz): 1.20 – 1.49 (38H, m, -CH_2-), 1.68 – 1.80 (8H, m, 4x ArO-CH_2-C$\underline{H}_2$-CH_2-), 3.95 – 4.05 (8H, m, 4x ArO-C$\underline{H}_2$-CH_2-), 8.67 (2H, ddd, *J* = 2.0 Hz, *J* = 2.4 Hz, *J* = 8.9 Hz, Ar$\underline{H}$), 6.91 (2H, ddd, *J* = 1.8 Hz, *J* = 2.1 Hz, *J* = 8.9 Hz, Ar$\underline{H}$), 6.95 (4H, ddd, *J* = 2.0 Hz, *J* = 3.0 Hz, *J* = 9.0 Hz, Ar$\underline{H}$), 7.01 (1H, ddd, *J* = 1.7 Hz, *J* = 8.0 Hz, Ar$\underline{H}$), 7.11 (1H, d, *J* = 1.7 Hz, Ar$\underline{H}$), 7.24 (1H, dd, *J* = 8.0 Hz, Ar$\underline{H}$), 7.44 – 7.56 (8H, m, Ar$\underline{H}$), 7.64 – 7.72 (8H, m, Ar$\underline{H}$)

^{13}C{^{1}H} NMR (100.5 MHz): 24.03, 24.40, 27.58, 27.0, 27.81, 27.87, 66.46, 109.18, 112.52, 113.33, 113.64, 117.50, 125.53, 126.12, 126.83, 128.72, 129.30, 131.13, 143.42, 149.23, 149.27, 158.21

MS M/Z (APCI): 973.5528 (calcd. for $C_{66}H_{73}N_2O_5$: 973.551400, M + H)

HPLC (RP): >99.5%

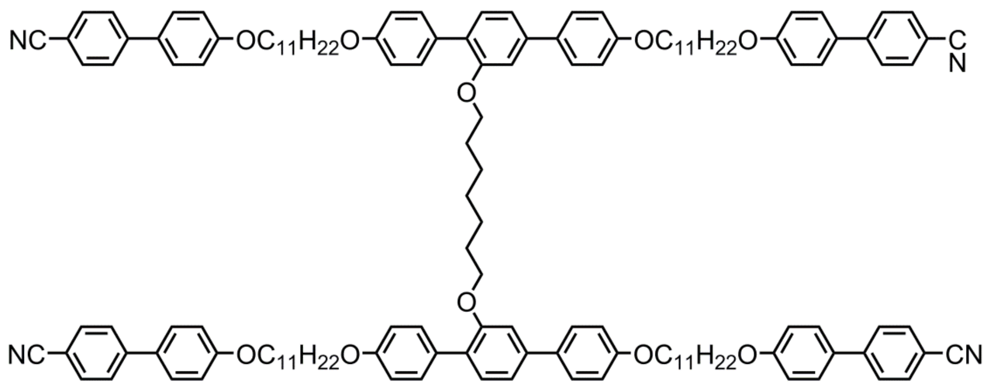

D11$_3$(2): 4',4''',4''''',4'''''''-(((((heptane-1,7-diylbis(oxy))bis([1,1':4',1''-terphenyl]-2',4,4''-triyl))tetrakis(oxy))tetrakis(undecane-11,1-diyl))tetrakis(oxy))tetrakis(([1,1'-biphenyl]-4-carbonitrile))

A suspension of **D11$_3$** (97.2 mg, 0.1 mmol), 1,5-dibromopentane (10.5 mg, 0.045 mmol) and caesium carbonate (1 mmol, 325 mg) in acetone (4 ml) was sealed into an Ace Pressure Tube and heated to 200 °C (external temperature) in a sand bath for 1h with vigorous stirring. The suspension was cooled, filtered and concentrated to dryness before purifying by column chromatography with a hexane:DCM/EtOAc gradient as the eluent. The chromatographed material was filtered through a 0.2 µm syringe filter before being recrystalised from ethanol twice to afford a white solid.

Yield: 49 mg (48 %)

Rf (DCM): 0.31 (fluoresces blue when irradiated at 254 & 365 nm)

^{1}H NMR (400 MHz): 1.20 – 1.51 (62 H, m, -CH$_2$-), 1.74 – 1.88 (20 H, m, -CH$_2$-), 3.97 (20H, m, ArO-CH$_2$-), 6.91 – 7.01 (18 H, m, Ar<u>H</u>), 7.40 – 7.55 (18H, m, ArH), 7.60 – 7.70 (18H, m, ArH).

^{13}C{^{1}H} NMR (100.5 MHz): 26.16, 26.19, 29.35, 29.44, 29.48, 29.51, 29.63, 29.67, 68.20, 68.30, 110.14, 114.14, 114.01, 114.85, 114.88, 115.21. 119.29, 127.21, 127.78, 128.19, 128.46, 130.62, 130.70, 131.37, 132.71, 133.43, 145.42, 158.35, 159.93

MS M/Z (APCI): 2042.1937 (calcd. for $C_{139}H_{156}N_4O_{10}$: 2042.1894, M + H)

HPLC (RP): >99.5%

3.1. Supplementary Photomicrographs

In order to demonstrate that the phase exhibited by the duplexed hexamesogen **D11$_3$(2)** is the TB phase we prepared a contact preparation with the well-studied bimesogen CB9CB, photomicrographs are presented in Figure SI-1 below. In (a) **D11$_3$(2)** is in the nematic phase at 150 °C while CB9CB is an isotropic liquid; in (b) both materials are in their nematic phase at 116 °C, in (c) and (d) **D11$_3$(2)** is in the TB phase whereas CB9CB is in the nematic phase; (e) was taken at the N-TB transition for CB9CB at 108 °C; (f) shows both materials are miscible in the TB phase at 104 °C. Based on this we conclude that **D11$_3$(2)** therefore exhibits the TB phase

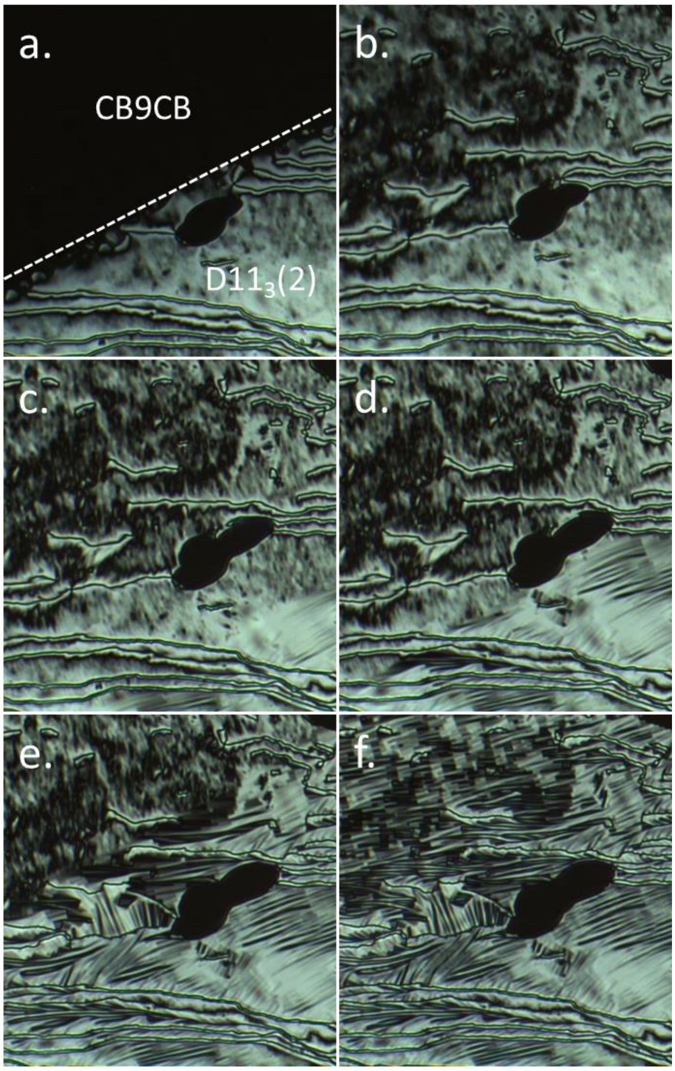

Figure SI-1: Photomicrographs (x100) of a contact preparation between CB9CB (top left) and the duplexed hexamesogen **D11$_3$(2)** (lower right): (a) 150 °C; (b) 118 °C; (c) 116 °C; (d) 110 °C; (e) 108 °C; (f) 104 °C. Each photomicrograph is of approximately the same area, note the air bubble (black).

References:

1. M. J. Frisch, G. W. Trucks, H. B. Schlegel, G. E. Scuseria, M. A. Robb, J. R. Cheeseman, G. Scalmani, V. Barone, B. Mennucci, G. A. Petersson, H. Nakatsuji, M. Caricato, X. Li, H. P. Hratchian, A. F. Izmaylov, J. Bloino, G. Zheng, J. L. Sonnenberg, M. Hada, M. Ehara, K. Toyota, R. Fukuda, J. Hasegawa, M. Ishida, T. Nakajima, Y. Honda, O. Kitao, H. Nakai, T. Vreven, J. A. Montgomery Jr., J. E. Peralta, F. Ogliaro, M. J. Bearpark, J. Heyd, E. N. Brothers, K. N. Kudin, V. N. Staroverov, R. Kobayashi, J. Normand, K. Raghavachari, A. P. Rendell, J. C. Burant, S. S. Iyengar, J. Tomasi, M. Cossi, N. Rega, N. J. Millam, M. Klene, J. E. Knox, J. B. Cross, V. Bakken, C. Adamo, J. Jaramillo, R. Gomperts, R. E. Stratmann, O. Yazyev, A. J. Austin, R. Cammi, C. Pomelli, J. W. Ochterski, R. L. Martin, K. Morokuma, V. G. Zakrzewski, G. A. Voth, P. Salvador, J. J. Dannenberg, S. Dapprich, A. D. Daniels, Ö. Farkas, J. B. Foresman, J. V. Ortiz, J. Cioslowski and D. J. Fox, *Gaussian 09*, 2009
2. M. Tarini, P. Cignoni and C. Montani, *Ieee T Vis Comput Gr*, 2006, **12**, 1237-1244
